# Recommendations and Improvements for the Evaluation of Integrated Community-Wide Interventions Approaches

**DOI:** 10.1155/2016/2385698

**Published:** 2016-12-26

**Authors:** Tessa M. van Koperen, Carry M. Renders, Eline J. M. Spierings, Anna-Marie Hendriks, Marjan J. Westerman, Jacob C. Seidell, Albertine J. Schuit

**Affiliations:** ^1^Department of Health Sciences and the EMGO Institute for Health and Care Research, VU University Amsterdam, Amsterdam, Netherlands; ^2^Department of Health Promotion, Faculty of Health, Medicine and Life Sciences, Maastricht University, Maastricht, Netherlands; ^3^National Institute for Public Health and the Environment (RIVM), Bilthoven, Netherlands

## Abstract

*Background*. Integrated community-wide intervention approaches (ICIAs) are implemented to prevent childhood obesity. Programme evaluation improves these ICIAs, but professionals involved often struggle with performance. Evaluation tools have been developed to support Dutch professionals involved in ICIAs. It is unclear how useful these tools are to intended users. We therefore researched the facilitators of and barriers to ICIA programme evaluation as perceived by professionals and their experiences of the evaluation tools.* Methods*. Focus groups and interviews with 33 public health professionals. Data were analysed using a thematic content approach.* Findings*. Evaluation is hampered by insufficient time, budget, and experience with ICIAs, lack of leadership, and limited advocacy for evaluation. Epidemiologists are regarded as responsible for evaluation but feel incompetent to perform evaluation or advocate its need in a political environment. Managers did not prioritise process evaluations, involvement of stakeholders, and capacity building. The evaluation tools are perceived as valuable but too comprehensive considering limited resources.* Conclusion*. Evaluating ICIAs is important but most professionals are unfamiliar with it and management does not prioritise process evaluation nor incentivize professionals to evaluate. To optimise programme evaluation, more resources and coaching are required to improve professionals' evaluation capabilities and specifically the use of evaluation.

## 1. Introduction

Worldwide, childhood obesity rates have increased over the last few decades [1]. Childhood obesity is related to a wide range of psychosocial and physical problems resulting in larger health care costs [2, 3]. Since obesity, once established, is difficult to treat, prevention is the main priority and is therefore receiving increasingly more attention.

Traditionally, the prevention and treatment of overweight and obesity have focused on stimulating changes in the behaviour of individuals [4]. Evidence showed that behaviours such as increased consumption of high energy density beverages and foods, low consumption of vegetables and fruits, less physical activity, and more sedentary leisure time activities contribute to overweight and obesity [5–7]. However, overweight and obesity develop within a sociocultural and physical environment in which these behaviours are made possible and sometimes even stimulated [8, 9]. So far obesity prevention efforts targeting individual behaviour [10] (e.g., education, treatment, and improving skills) have unfortunately had limited effects [11, 12]. Therefore, interventions at a higher level are needed [13–15]. Since overweight and obesity are caused by multiple interacting determinants, it is important to address both distal and proximal determinants of obesity [16–18] using an “integrated community-wide intervention approach” (ICIA). This approach aims to conduct multiple interventions, in a collaborative effort with multiple stakeholders, that work in conjunction with each other in multiple settings and are directed towards multiple target groups in a community [12, 18–23]. Such approaches are believed to be more effective because they target multiple individual and environmental determinants of obesity.

An exemplary ICIA is the French EPODE programme (“Ensemble Prévenons l'Obésité De Enfants” or “Together Let's Prevent Childhood Obesity”). EPODE aims to prevent overweight and obesity in children aged 0–12 years and their families through a multiactivity, multisetting, and multistakeholder approach [24, 25]. This comprehensive approach is coordinated at central level. The focus is on promoting healthy behaviours related to overweight and obesity, for example, healthy eating and regular physical activity through multiple organised activities to the children, their families, and intermediaries [24–26]. At the community-level, a programme manager is nominated by local authorities. This programme manager not only is trained by the central coordination team but also is provided with tools and instruments facilitative to local implementation [24, 25].

In Netherlands, the nationally coordinated ICIA “Youth on a Healthy Weight” (In Dutch: Jongeren Op Gezond Gewicht, with the acronym “JOGG”) has been implemented in more than 100 municipalities since 2010. This approach is based on the EPODE programme. The JOGG-approach is based on five critical components or “pillars”: (1) political commitment, (2) public-private partnerships, (3) use of social marketing principles, (4) scientific evaluation and dissemination, and (5) linking prevention and healthcare [25, 27, 28].

To improve an ICIA such as the JOGG-approach, evaluation is indispensable. In this paper, the definition of programme evaluation refers to the systematic and objective assessment, analysis, and reporting of information on an on-going programme. It starts with the design and follows the implementation of the programme in order to understand the process and impacts of interventions, to make it possible to adapt the programme given interim findings and to inform decision-making. When started simultaneously with the implementation of an ICIA, evaluation can increase stakeholder and community participation and provide information allowing programme managers to improve the programme [29]. Furthermore, it can increase accountability, as well as strategic and financial support, and enhance the sustainability of resources [30]. Moreover, programme evaluation may assist in the dissemination of knowledge of the programme and of elements that are proving successful to other areas and professionals [11, 30]. Although important for programme success, the evaluation of ICIAs is challenging. ICIAs are complex interventions, and as a result their evaluation is also complex [31–34]. This complexity demands evaluators to go beyond the traditional notions of evaluation research and experimental design [35–38]. It also demands that the evaluation be tailored to the characteristics of a specific ICIA, to the needs of evaluation users and stakeholders, and to the expected outcomes [39–41].

Professionals who are responsible for the programme evaluation of their ICIA often struggle to carry out such evaluation satisfactorily [42–44]. Within the JOGG-approach, these professionals are the programme managers (usually policymakers from local government) or epidemiologists (employed by the Regional Public Health Services (RPHS)). In order to assist them with programme evaluation of the JOGG-approach, evaluation support tools have been disseminated by the National Coordination Office of the JOGG-approach (JOGG-office). These evaluation tools consist of an Evaluation Manual and additional evaluation training [45]. The Evaluation Manual describes a six-step evaluation process for programme evaluation of ICIAs, similar to the CDC Evaluation Framework [46] and the outline for evaluating an initiative by the Kansas University Community Toolbox [47]. The design of the Evaluation Manual is based on the outcomes of a comprehensive appraisal of existing evaluation frameworks [48]. It also provides a logic model for the JOGG-approach (the JOGG-model), capacity building exercises, an evaluation planning matrix, checklists, and examples of evaluation practice. The evaluation training consists of four modules following the evaluation approach as described in the Evaluation Manual.

The reasons for this study were the outcomes of the pretraining assessment forms and discussions with programme managers and epidemiologists involved in the local JOGG-approaches in 2012 and 2013 during their evaluation training. These revealed that the evaluation budget for most new JOGG-municipalities was zero. Moreover, programme managers and epidemiologists participating in the evaluation training reported having to continuously juggle their desire to customise evaluation to their local context, needs, and assets, which necessarily costs time, effort, and money on the one hand, with their need to standardise the evaluation using existing datasets, monitors, and standard evaluation instruments (since hardly any or indeed no evaluation budget was available) on the other hand. As a consequence, only six of the 60 local JOGG-approaches had developed evaluation plans by the end of 2013. We concluded that despite support given (i.e., Evaluation Manual and evaluation training), there were a lot of barriers to programme evaluation of the local JOGG-approach. The aim of our study was therefore twofold: (1) to explore barriers to and facilitators of programme evaluation of ICIAs and (2) to understand the experiences of programme managers and epidemiologists regarding the offered evaluation tools.

## 2. Methods

### 2.1. Study Design

A qualitative study was conducted using a framework approach to collect data on both aims of this study. For the exploration of barriers to and facilitators of the programme evaluation of ICIAs, the “Behaviour Change Wheel” from Michie et al. [49] was used to guide semistructured interviews and their analysis. For the second aim, experiences of the evaluations tools, we used the innovation characteristics from the “Diffusion of Innovation” theory by Rogers [50, 51] to guide focus groups and to analyse our findings.

### 2.2. Recruitment and Sampling

A purposeful sampling technique was used to recruit participants for the semistructured interviews and the focus groups [52].

#### 2.2.1. Semistructured Interviews

**Figure 1:**
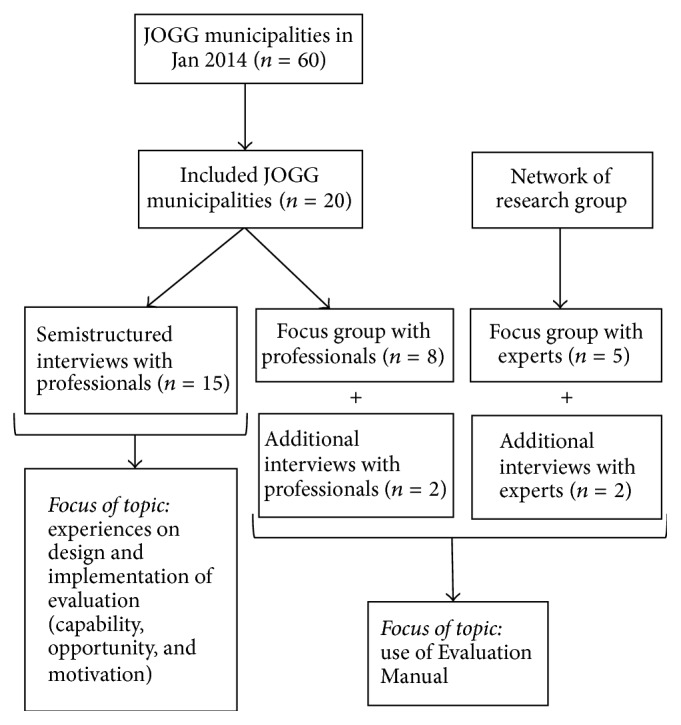
Graphic of study sampling.

For the semistructured interviews, participants were recruited from municipalities who had been involved in the JOGG-approach for at least one year (*n* = 20) (Figure 1). This criterion was chosen to ensure that they had sufficient experience with the JOGG-approach and in the hope that participants would provide experiential knowledge. The participants were programme managers and epidemiologists involved in the JOGG-approach. Due to the already complex intervention approach, we choose to include representatives from JOGG-municipalities that had one programme manager and one involved epidemiologist. This decision was taken because responsibilities were then clearer. Participants were invited by email and arrangements for conducting the interviews were made by phone. The final sample consisted of eight programme managers and seven epidemiologists from nine different municipalities.

#### 2.2.2. Focus Groups

Two focus groups were held (Figure 1). One consisted of programme managers and epidemiologists involved in JOGG-approaches that had commenced at least one year ago (FG1). The other focus group consisted of experts in the field of community health promotion and evaluation (FG2). To recruit participants for FG1, an invitation letter was sent to the programme managers and epidemiologists involved in JOGG-approaches. Additionally, the JOGG-office sent out an announcement email to JOGG-municipalities with a participation request. Participants for FG2 were recruited from the network of the research group and were only included if they were* not* responsible for the evaluation of local JOGG-approaches. Focus group participants were afforded travel allowance and a €10 gift card. If participants did not respond, we called them one week after the invitation was sent. If participants failed to respond after three reminder calls, we excluded them from the study. When participants could not attend the focus groups (e.g., due to time limitations), we suggested an interview (*n* = 4). The final sample consisted of ten professionals for FG1 and the additional interviews (i.e., three programme managers and five epidemiologists) and seven experts for FG2 and the additional interviews.

### 2.3. Interview Guide and Topic List 

#### 2.3.1. Semistructured Interviews

The first author (MVK) and two assistant researchers (RH and EB) together developed the interview guide for the semistructured interviews (see Supplementary Data 1 in Supplementary Material available online at http://dx.doi.org/10.1155/2016/2385698). This interview guide was based on the “Behaviour Change Wheel” [49] and was extended with questions regarding “suggestions for improvements” and “needs with respect to the programme evaluation.” A central component of the “Behaviour Change Wheel” is the “COM-B” system: Capability, Opportunity, Motivation, and Behaviour. To illustrate, evaluation (behaviour) is enabled if the target populations (e.g., programme managers) have sufficient understanding on how to apply the Evaluation Manual (capability); have sufficient time and financial means to apply the Evaluation Manual (opportunity); and have a positive attitude towards using an Evaluation Manual (motivation).

#### 2.3.2. Focus Groups

The first author (MVK) and one assistant researcher (ES) together developed the topic list for both focus groups and the additional interviews (Supplementary Data 2). Rogers' theory, the “Diffusion of Innovation,” was used as an initial organising framework to generate topics [50, 51, 53]. Rogers (2002) assumes that innovations (e.g., the Evaluation Manual) are more likely to be used if (1) they can easily be tried (in this case, if the Evaluation Manual can be used without major changes or consequences in the organisation); (2) the relative advantage of the innovation is high (if the use of the JOGG Evaluation Manual requires less time than current procedures or Evaluation Manuals); (3) the innovation is compatible with the daily practice of users (i.e., those responsible for evaluation); (4) the innovation is not complex (if Evaluation Manual is perceived to be easy to use); and (5) the outcomes of using the innovation can be observed (if use of the Evaluation Manual leads to better goal setting or alignment of stakeholder needs) [50, 53]. Since the experts had not been working with the Evaluation Manual, the focus of their group discussion was not based on their own experience but on their perception of use of the Evaluation Manual for programme managers and epidemiologist in general.

### 2.4. Data Collection

#### 2.4.1. Semistructured Interviews

The semistructured interviews were conducted by RH (with programme managers) and EB (with epidemiologists) between March and June 2014 at a convenient time identified by the interviewees. Open-ended questions were used to give participants the opportunity to share their experiences on design and implementation of evaluation in their own words [54]. Prior to data collection, all respondents were asked to sign an informed consent form. Interviews lasted on average 53 minutes (with a range within 27–68 minutes) and were audiotaped. In order to check our interpretation of the semistructured interviews, the respondents received a summary of their interview and were asked whether they agreed with our interpretation or wanted to change or add anything (member check).

#### 2.4.2. Focus Groups

The focus groups and the additional interviews took place between January and February 2014. The focus groups were conducted at a central location and the additional interviews took place at a convenient time and location identified by the interviewees. Two weeks before the focus groups and interviews, all participants received the Evaluation Manual and prior to the data collection all signed an informed consent form. Focus groups were conducted by an experienced independent moderator (MK) and an assistant moderator (ES). Each focus group took 120 minutes and was audiotaped. Four professionals not able to attend the focus groups were subsequently interviewed. The interviews lasted on average 67 minutes (with a range within 53–120 minutes) and were audiotaped and transcribed verbatim by the interviewer (ES).

### 2.5. Data Analyses

The transcripts from the semistructured interviews, the focus groups, and the additional interviews were thematically analysed using open and axial coding and divided between the main themes from the respective theoretical frameworks used. This helped to structure and identify reoccurring themes [55]. After this separate data analysis, data was merged where it provided additional information or overlapped data within the other framework (i.e., focus group data regarding “feasibility of use of the evaluation manual” was merged with “perceived opportunities to conduct programme evaluation” within the Michie theme “Opportunity”; focus group data regarding “complexity of the evaluation manual” was merged with “perceived barriers and facilitators to evaluate” within the Michie theme “Motivation”).

#### 2.5.1. Semistructured Interviews

Fragmentation and open coding of the first transcript were done by the interviewers (RH; and EB) and discussed with the first author (MVK) and the fifth author (MW). Subsequently, axial coding was used to code fragments in subcategories or main categories. Identified categories were divided between the main themes from the “Behaviour Change Wheel” [49]. This process resulted in a code tree that was then used to code the other interviews (Supplementary Data 3). The themes were the basis for describing the barriers and facilitators that programme managers and epidemiologists experienced with programme evaluation and the use of the evaluation tools.

#### 2.5.2. Focus Groups

In the open coding phase, two researchers (ES and MVK) independently read the transcripts of the focus groups and interviews and coded fragments. This resulted in memos and an inductive code list with in vivo codes which were subsequently discussed. Axial coding was then used to code fragments in sub- or main categories (ES) (Supplementary Data 4). During this iterative process, new relevant themes were added to the code list. Finally, both researchers conducted selective coding by summarizing the focus groups and the additional interviews. This gave more insight into explanations and causal relations within the data.

## 3. Findings

### 3.1. Description of Sample

The municipalities started the JOGG-approach between 2009 and 2013. Five programme managers and four epidemiologists were involved from the start of their JOGG-approaches. Of the interviewed programme managers, only one was experienced with ICIAs and none had been involved in the evaluation of an ICIA (Table 1). Two of seven epidemiologists were experienced with ICIA evaluation. Approximately 65% of the programme managers attended less than 2 training sessions in evaluation and none attended all four sessions. Reasons given for this limited attendance were the limited hours a week available for the JOGG-approach besides other responsibilities. Programme managers had approximately 16 employable hours available per week for the JOGG-approach, within these hours evaluation was not a priority and training sessions took too much of their available time during a week. More than half of the programme managers had other responsibilities, such as being a policymaker for the sport or health sector at the municipality or being a public health professional at the RPHS. The epidemiologists were better represented at the evaluation training, more than 70% attended more than three (out of four) training sessions, although most had less hours available per week for the JOGG-approach than the programme managers. They either felt responsible for the evaluation of the JOGG-approach or were sent by their programme manager. Two epidemiologists worked for multiple JOGG-municipalities.

The epidemiologists in focus group 1 were all employed by the RPHS, the programme managers within the municipality. The experts in focus group 2 were three senior researchers in the field of public health from three universities, two entrepreneurs at strategic level in public health, a senior researcher from a research institute specialised in overweight, and a programme evaluation expert employed at a University.

### 3.2. Evaluation Performance within JOGG-Municipalities

Programme evaluation is “the systematic collection of information about the activities, characteristics, and outcomes of programmes to make judgments about the programme, improve programme effectiveness, and/or inform decisions about future programme development” [56]. In general, it consists of a process and an effect evaluation for which an evaluation plan has to be written. Since the JOGG-approach needs to be adapted to local context and the needs of stakeholders, and available resources, the design and implementation of the JOGG-approach differed between municipalities and so does the programme evaluation.

Most interviewees referred to four types of evaluation that they conducted for their JOGG-approach: (1) a* process evaluation* that focused on organised activities, (2) a* process evaluation* that focused on the quality of the collaboration with implementing partners, (3) an* intermediate evaluation* of behaviour change in children, and an (4)* effect evaluation* of overweight in children. All but one of the programme managers and epidemiologists conducted either one or a combination of these evaluations. In one municipality, an integrated approach preceded the JOGG-approach, commencing in 2008. Respondents from this municipality showed more capability, conducted multiple evaluation types, and had more resources than other municipalities that started the JOGG-approach in the same year. Ideally, programme managers should be engaged in evaluation to ensure a common understanding of the purpose and scope, to properly budget for the evaluation activities, and to clearly assign roles and responsibilities to programme stakeholders [45]. Although most programme managers were involved in the development of an evaluation plan and some were involved in data analysis, they were never involved in data collection. Often programme managers did not consider it their task to coordinate evaluation (let alone evaluate themselves) and felt no urge to fulfil these roles. For example, one programme manager completely delegated evaluation to the RPHS and to university students; she did not know who were involved with the evaluation of “her” programme, what their tasks were, what they were planning to measure, and if they aligned their evaluation to the programme:

*“She [name of epidemiologist] is responsible for the research […]. Moreover, I believe that the programme manager should not have knowledge of all these things [research and evaluation]”* [RI21]

*“I need to align with the researcher to understand how we are going to use it [the establishment of the Impact Assessment], because she is more involved in it than I am.”* [RI27]

This perception of responsibilities in evaluation and lack of role fulfilment may explain why programme managers were often not participating in the evaluation training and why one epidemiologist (RI9) said that the evaluation work “group” consisted of one person (herself). As a result there was no shared responsibility for evaluation and motivation to evaluate decreased:

*“It remains a bit tricky when no one feels responsible for it [monitoring and evaluation]. And so, who is going to complete the activity monitor? And who then makes sure that all the data will be collected and inserted?”* [RI29]

Epidemiologists were mostly involved in an intermediate evaluation of behaviour change or an effect evaluation of overweight in children in which they conducted baseline measurements, follow-up data collection, and data analysis.

### 3.3. Barriers to and Facilitators of Programme Evaluation

#### 3.3.1. Knowledge and Skills

Most epidemiologists and programme managers were struggling when they attempted to conduct a programme evaluation within ICIAs because they were only familiar with outcome evaluations:

* “This [the evaluation of the JOGG-approach] is a different kind of evaluation and research than what I'm used to, I'm not experienced in doing this and I feel I really miss this expertise.” *[RI26]

Evaluation was further complicated because implementing an ICIA like the JOGG-approach itself was new for most programme managers and epidemiologists (Table 1). Some interviewees therefore said they required different or more specific evaluation support (e.g., measurement instruments, indicator overview, budget calculations, information on how to conduct qualitative research, and how to set up a community intervention trial):

*“I expected more of the JOGG evaluation training […] like, this is what the evaluation looks like and this is the data you need to collect, and this is how we are going to do it”* [RI29]

A majority of epidemiologists understood that setting concrete evaluation goals for their ICIA was important. However, most believed this to be difficult, as they did not want to be accountable for such specific and unattainable goals:

*“We also set milestones, like the percentage of kids that should exercise, or 10% of our primary schools this year will have a healthy policy. And that is what we measure."* [RI21]

*“[…] for that we still haven't formulated a specific objective *…* it remains truly difficult to be presented with a bill for goals that appear not to be feasible in the end.”* [RI31]

Some programme managers clearly made use of the Evaluation Manual; they involved external parties (e.g., primary schools and a health centre) to set such goals and made agreements about who would actually implement, develop, and conduct the evaluation of these goals:

*“We started with a meeting for all stakeholders [for the evaluation planning] to create support. In this meeting we set goals and objectives … for processes, behaviour and outcomes … people could highlight the ones they believed were most important.”* [RI25].

In line with this, several programme managers emphasised that clear communication about evaluation towards stakeholders (e.g., RPHS, epidemiologists, employees of a sport-centre, aldermen, and school directors) was important (Table 2). They said that clearly explaining the relevance and goals of evaluation and aligning evaluation efforts were most important. However, some programme managers admitted they did not always invest in such communication resulting in less attention to all aspects of programme evaluation, for instance, to process evaluation:

*“I have to say that the process evaluation received less and less attention. Although we had made really clear arrangements and activities of what we were going to do. And now, the process evaluation has faded into the background.”* [RI26]

#### 3.3.2. Support and Finance for Programme Evaluation

Programme managers said the opportunity to evaluate improved if it was possible to discuss the evaluation with a group or person who felt responsible for evaluation. They struggled with taking responsibility to start the evaluation. This apprehension caused them to consider collaboration with an epidemiologist, researcher, or evaluation work group. Programme managers felt this collaboration to be crucial for the performance of programme evaluation of ICIAs (Table 2). Additionally, availability of students, volunteers, activity coordinators, and public-private partners was considered important, since they often collected data for the evaluation. In this way, the programme managers could save time and financial resources:

*“The university has said it would like to contribute to the evaluation of JOGG with X number of students. And of course this includes some support from the university.”* [RI23]

Respondents mentioned that financial resources often imposed a barrier since municipalities had just faced drastic budget cuts and decentralization of tasks to municipalities. Moreover, time was a barrier when programme managers needed to invest in new activities and explain the integrated approach to stakeholders (i.e., when the JOGG-approach was not based on an existing ICIA), when limited professionals were available for the JOGG-approach at local level, when no time was allocated for evaluation (i.e., some epidemiologists were employed for the JOGG-approach for 2 hours a week), or when time was used inefficiently due to lack of professionals with experience in the evaluation of ICIAs. One programme manager (RI23) (who was employed for 18 hours a week to the JOGG-approach) explained this was insufficient to manage such a comprehensive programme with high expectations:

* “JOGG itself states that a half FTE for a programme manager is sufficient. I have my doubts.”* [RI23]

Most epidemiologists thus considered it important to show the amount of time that was needed for a proper evaluation:

*“It is especially prior to conducting an evaluation that you should take the time and talk to each other about ‘What information do we track?', ‘What do we want to register?', ‘Who feels responsible?' and ‘Who is going to do it?' and this all takes quite some time.”* [RI29]

Additionally, one epidemiologist said that even though 10 hours were available for evaluation, he felt these were used inefficiently due to lack of experience; more time was needed to get acquainted with the programme evaluation process. Especially in smaller municipalities, sufficient time to conduct all sorts of evaluation was considered unfeasible. Epidemiologists considered programme managers to be responsible for obtaining more financial resources to make evaluation feasible and added that when a municipality considers it important to evaluate, budget is made available. Contrastingly, several programme managers stated it was extremely hard to influence what level of resources would be made available from the municipal budget and allocated to process evaluation. To make evaluation more feasible, programme managers from smaller municipalities suggested the organisation of programme evaluation at a regional level and the alignment of data collection. In this way evaluation expertise, resources, and tools (e.g., questionnaires) could be shared:

*“*…* I know that [a baseline in a sample group] is far preferable to group data [the whole population] but we just do not have time for it. So, we will say that ‘now N percent of the target group drinks water' and next year ‘so much percent will drink water'. And that is enough for us and for the local government.”* [RI33]

To stimulate the sharing of evaluation responsibility, it was considered important that external partners had a stake in evaluation. For example, teachers and directors of primary schools were often interested in diet and physical activity and overweight of “their” children, and therefore often agreed to participate in evaluation. Some programme managers indicated finding such stakeholders was difficult when the JOGG-approach was poorly understood:

*“Sometimes that penny [to know what the JOGG-approach is] just has not dropped yet *…* I'm not the JOGG-approach, but they are! The stakeholders, the ultimate target group, the community, they are the JOGG-approach!”* [RI22]

Moreover, collecting data among 12–18 year olds was considered difficult since these youngsters were overwhelmed by surveys, and schools were sometimes concerned about their privacy. Similarly, private partners were not always willing to contribute financially to evaluation or to participate in conducting a programme evaluation. Additionally, student or private partners sometimes gathered evaluation data of insufficient quality:

*“Quite often I get handed rattled off pieces, obviously motivation is lacking.”* [RI21]

#### 3.3.3. Motivation of Programme Managers and Epidemiologists to Perform Programme Evaluation

The main motivations of programme managers and epidemiologists to conduct an evaluation were as follows: interest in childhood obesity prevention and ICIA; seeing evaluation as a natural part of the work process; increasing accountability for the JOGG-approach towards the aldermen and council; understanding of why the JOGG-approach is effective; motivation of stakeholders to participate in the JOGG-approach; securing of resources for the JOGG-approach; and the opportunity to present the results on a national level and compare their progress with other municipalities (Table 2):

* “… It's just really fun to work on a project which is doing good and has a national reputation. If the results are called at the national JOGG-conference, than it is very nice to hear that our JOGG-approach is doing so well. It is often very hard work and we slog at it but it still very stimulating*.” [RI22]

*“*…* they [strategic and tactical managers] would occasionally like to have numbers, they want to show that the programme works and they want to know whether it works.”* [RI19]

Programme managers and epidemiologists were demotivated by perceived lack of the necessary knowledge and skills to conduct an evaluation:

*“The process evaluation, I find it extremely difficult *…* so it actually is a kind of delay although I do see that's very important.”* [RI26]

Another aspect that can cause a lack of motivation to evaluate is the strong interest of tactical management (i.e., aldermen) and strategic management (i.e., department or sector manager) in the implementation rather than the evaluation of the ICIAs. Especially in smaller municipalities, interviewees considered it not worth their while to shift scarce resources away from implementation to conduct process, intermediate, or effect evaluations. Also programme managers explained that it did not make sense to start evaluation if activities had not yet been implemented, which epitomises their (lack of) understanding of the importance of programme evaluation to the optimisation of programme:

*“I reason very much in the interest of my people and my people have no interest in such monitoring, they have an interest in policy measures, activities, incentives, whatever … so that is my priority.”* [RI28]

*“I can now emphatically focus on evaluation, but if we don't do anything, then I actually do not need to evaluate, so the focus is on performing and doing.”* [RI23]

Although, in numerous cases, no budget was allocated for evaluation in any form, where municipalities were interested, they tended to prioritise effect evaluation.

*“… [We noticed] that they [managers and aldermen] would occasionally like to have numbers, they want to show that the programme works and they want to know whether it works.”* [RI29]

As a result, programme managers were less motivated to carry out other types of evaluation:

*“*…* so that component [the effects] I can measure *…*, we report about this as well, which is why we have determined this our priority.”* [RI21]

### 3.4. Use and Expectations of the Evaluation Manual

Most programme managers and epidemiologists knew the Evaluation Manual but not a single one of the respondents had used it as intended. Despite this limited use, most respondents were positive about the existence of the Evaluation Manual; it was considered a relevant and valuable innovation to support planning and performance of an ICIA programme evaluation. Experts said the Evaluation Manual was relevant because they see programme managers and epidemiologists struggle with the evaluation of ICIAs. Several reasons for limited use of the Evaluation Manual were mentioned: almost all respondents said the Evaluation Manual was too comprehensive; following the steps in detail would definitely exceed the proposed evaluation budget as mentioned in the Evaluation Manual (10–15% of total programme budget) or available time; most programme managers and epidemiologists said their financial resources were insufficient to even implement the ICIA, and therefore they could not find the time needed for evaluation or could not use their time for evaluation purposes:

*“I have 3 hours a week for two municipalities.”* [RF1]

*“If a municipality does not recognize the importance of it [conducting the evaluation following the Evaluation Manual] then it does not happen.”* [RI32]

However, experts also reasoned that evaluation budget could easily be obtained from funders (i.e., municipality, grants), if programme evaluation had been included in the initial planning of the ICIA and requesting funding for it.

Another reason offered for limited use of the Evaluation Manual was that some parts were considered to be more useful than others, but opinions on this differed. Some respondents were positive about a graphic illustrating growth and achievement of programme goals in a time-line saying it helped them to manage the expectations of stakeholders. Others said that the guiding notebooks presented in the Evaluation Manual helped them to process theory into practice. Some programme managers and epidemiologists found the JOGG logic model especially relevant, while others said it was more facilitative to develop their own logic model. Furthermore, experts suggested the logic model could be used as a guideline for the planning of programme evaluation in addition to the six-step evaluation roadmap:

*“I would recommend using evaluation planning following the JOGG-model and maybe less strict through the six-stepped roadmap. And to determine evaluation type from goals on different levels, and provide examples to make this concrete.”* [RF9]

All respondents added that the main reason for only using parts of the Evaluation Manual was that reading the Evaluation Manual was too time-consuming for professionals at the operational level. Another reason mentioned for this limited use was that it was difficult to use the suggested evaluation approach in an ICIA that had already started and was in an implementation phase. Since the Evaluation Manual started by defining stakeholders and collaboratively setting goals and objectives the respondents felt as if they were going back to the drawing board if they had to follow the Evaluation Manual. They wanted to progress and did not see the advantage of a new approach to a programme which had already been approved by funders and management. Another reason for limited use was that the evaluation approach was difficult to align with the emphasis of the council or aldermen on effect evaluation:

*“The town council wanted to see a decrease in BMI at any price […] the alderman's' head is on the block for it.”* [RF1]

Although some aldermen were open to discussion and liked to have expectations managed,

*“he wants to know that stakeholders are involved in the right way, those stakeholders are important to him and to the programme.”* [RF2]

All respondents said the Evaluation Manual could be very useful but that the evaluation approach proposed in the Evaluation Manual was too linear, theoretical, and comprehensive for daily practice. To improve the Evaluation Manual, experts suggested emphasising the involvement of stakeholders in programme design and evaluation, underscoring the importance of synchronising ambitions and expectations of several stakeholders in the community and expressing bottom-up strategies with examples in order to involve the community.

## 4. Discussion

Our main findings indicate that those responsible for programme evaluation, programme managers and epidemiologists, often lack specific* knowledge and skills* to conduct a programme evaluation (capability) in its fullest, perceive* limited time and financial resources *(opportunities) to conduct a comprehensive programme evaluation, and are seldom* internally or externally motivated *(lack of incentives) to conduct a programme evaluation. We also found that professionals involved in ICIAs do not often use the Evaluation Manual provided and that evaluation training was poorly attended, especially by programme managers. We will now discuss these findings and recommend ways to improve programme evaluation.

First of all, even though programme managers understood the importance of evaluation, they did not invest in its management. A perceived lack of time and role related expectations may explain this. Programme managers often felt forced to invest time either in implementation or in evaluation and often prioritised implementation. If they were investing time in evaluation, they focused on assessing outcomes rather than processes. Programme managers did not see reflection and adaptation, as part of a process evaluation, as ways to continuously improve their ICIA. Insufficient programme evaluation knowledge and skills seem to explain the limited involvement of programme managers [41, 42, 57]. Besides insufficient skills, role related expectations seem to explain the limited involvement of these managers. Often epidemiologists were seen as evaluation experts and therefore regarded responsible for evaluation. Unfortunately, epidemiologists felt unable to fulfil this role since programme evaluation of ICIAs was new to them. Even though they were familiar with quantitative outcome and impact evaluations, they required new skills and support (e.g., guidance and management) to conduct a process evaluation (as part of a programme evaluation). This need for more technical evaluation support was also felt by others [30, 42, 57–59].

Secondly, time, manpower, and money were insufficiently allocated to evaluation and generating funding (i.e., budget) for evaluation proved difficult. One important reason for poor generation and allocation of evaluation resources was related to insufficient knowledge; professionals, and especially programme managers, often did not know* what* and* how* they had to evaluate and seemed unaware that evaluation requires management and guidance. Additionally, some professionals did not see the benefits of programme evaluation; they perceived more cons than pros (e.g., taking too much money and work). This is in line with Torres and Preskill (2001) who found that professionals were concerned that evaluation would lead to undesirable results (e.g., dissatisfaction of capability) and therefore have a reduced motivation to engage in or manage the evaluation. On the contrary, we also found some professionals, especially epidemiologists, motivated to evaluate; these were mostly epidemiologists who liked the idea that it was “new” to work within ICIA, who believed evaluation was important for the ICIA or had personal interest in (reduction of) childhood obesity prevention. Unfortunately, these motivated professionals were often demotivated by strategic and tactical managers allocating scarce resources to the integrated community-wide intervention approach and showing more interest in implementation than evaluation. This perception of interest in implementation rather than evaluation was also supported by others [42]. Another reason for limited evaluation resources is the lack of public-private partnerships at the local level. When private parties finance activities, the municipal budget can be allocated for program management and evaluation. When private parties are not involved, the ICIA budget was mainly allocated to the implementation of activities.

Thirdly, the lack of time, role related expectations, and poor allocation of evaluation budget also contributed to the limited use of the Evaluation Manual and may explain why programme managers scarcely visit evaluation training. Although the Evaluation Manual was seen as a useful tool for more concrete and practical programme evaluation, it was considered too comprehensive (i.e., taking too much time) and linear for practical use. Moreover, stakeholders perceived programme accountability as achieved through assessing effects at health outcome level, rather than a full programme evaluation. This may also explain why only parts of the comprehensive Evaluation Manual were used.

### 4.1. Recommendations for Practice

Firstly, we recommend increasing skills and knowledge of programme managers and epidemiologists. They need to learn how to plan, conduct, and advocate programme evaluation and involve stakeholders throughout the evaluation process. Learning these new skills can be stimulated through providing technical support for the design and implementation of the evaluation and an Evaluation Manual with multiple stratified versions as well as a stronger emphasis on stakeholder involvement. An evaluation training should be made mandatory for programme managers and epidemiologists and provided at the municipality and supported by strategic and tactical managers of the municipality. The Evaluation Manual can provide direction during or structure to the evaluation training.

Secondly, we recommend making clear agreements as to the roles and responsibilities between stakeholders in the ICIA evaluation. Programme managers should realize that evaluation requires their guidance and management and they should take charge of the planning and performance of the programme evaluation. Epidemiologists should lead outcome evaluation, establish the evaluation methodology and measurement instruments, and involve stakeholders (e.g., defining needs and resources). Policy makers could be involved in the evaluation of the municipal structure and organisation and specify the municipal evaluation budget. Therefore, increased communication between all stakeholders is of crucial importance.

Thirdly, we recommend investing in convincing strategic and tactical managers that the results or benefits of evaluation outweigh the costs. Programme managers and epidemiologist can, for example, emphasise that evaluation is useful for programme improvement and has positive consequences for programme continuation and that these outweigh the costs related to planning and performing programme evaluation [49, 60].

Fourthly, we recommend ensuring that resources are allocated to evaluation. This can be achieved by involving private stakeholders and making an explicit decision to generate and allocate evaluation resources* before* designing the evaluation [61]. Therefore, we recommend that programme managers communicate the evaluation plan and the necessity of programme evaluation to* all* stakeholders and ensure that roles are clearly divided between them. When multiple stakeholders are involved in planning and conducting the programme evaluation, it is more likely that resources and necessary preconditions for programme evaluation will be put in place.

Fifthly, municipal organisation is recommended to increase the dialogue on evaluation. This might be achieved through standardising programme evaluation in policy documents and establishing evaluation workgroups. The JOGG-office can stimulate this dialogue by requesting a specific evaluation budget attached to the municipalities' ICIA programme description. They should also discuss the relevance of programme evaluation for the success of the JOGG-approach in the first exploratory talks between a JOGG account manager from the JOGG-office and tactical manager who will decide whether or not the municipality will participate in the JOGG-approach.

### 4.2. Strengths and Limitations

The strengths of this study are the open character of the interviews, member checks, and heterogeneity of our data. Although respondents knew they would be interviewed on the evaluation of JOGG, and some received questions prior the interview, they seemed comfortable with providing sensitive data and were not inclined to give socially desirable answers [52]. Moreover, since respondents had different roles in ICIAs and represented both relatively small and large municipalities and five different provinces, we obtained a panoramic view on facilitators and barriers. A limitation of the study is that theoretical saturation was not reached due to the small number of respondents and the context in which the programme manager and epidemiologist worked was not explicitly taken into account. Additionally, not one of the respondents had used the Evaluation Manual as intended which limited their full understanding of its use when conducting a programme evaluation.

## 5. Conclusion

Programme evaluation is an important element of ICIAs such as JOGG but is often omitted in the current complex policy and political environment. Evaluation is often regarded as too comprehensive or focused purely on outcome evaluation. Furthermore,* implementation* rather than* evaluation* absorbs most resources. Additionally, evaluating ICIAs is new for many professionals and not well managed. In this context, an Evaluation Manual seemed insufficient to stimulate programme evaluation. Usefulness of an Evaluation Manual might improve by prioritising evaluation, providing evaluation incentives, and encouraging municipalities to adjust organisational preconditions. Additionally, bottom-up strategies such as involvement of the community and synchronising ambitions and expectations of stakeholders are necessary. Assigning a coach to the local ICIA organisation may be a valuable way to implement these bottom-up strategies. This evaluation coach should support the programme manager and the epidemiologist in involving the community (target group and stakeholders), to raise awareness of the importance of evaluation at operational, tactical, and especially the strategic level (for obtaining resources) and give support by applying the Evaluation Manual in the local context given available resources.

## Supplementary Material

The Supplementary Material comprises of two instruments used for data collection and two developed tools for the analysis. Supplementary Data 1 is the interview guide for the semi-structured interviews with operationalized concepts from Michie's Behaviour Change Wheel [49]. Supplementary Data 2 is the topic list for the focus groups and additional interviews regarding the Evaluation Manual, based upon the innovation characteristics of Roger's Diffusion of Innovation Theory [50, 53]. Supplementary Data 3 is the code tree for the axial coding of the fragments of the semi-structured interviews and Supplementary Data 4 is the code tree for the axial coding of the fragments of the Focus Groups.

## Figures and Tables

**Table 1:** Study sample.

Semistructured interviews							Focus groups		
	Sex^*∗*^	Occupation^*∗∗*^	Employed for JOGG (hours per week)	Experienced with ICIA	Experienced with ICIA evaluation	Attendance to JOGG evaluative training		Sex^*∗*^	Occupation^*∗∗*^
							*Focus group 1: JOGG professionals*		
RI21	F	P	±20	No	No	1	RF1	F	E
RI22	F	P	±24	Yes	No	3	RF2	F	P
RI23	M	P	±18	No	No	1	RF3	M	E
RI24	F	P	±4	No	No	1	RF4	F	E
RI25	F	P	±24	No	No	3	RF5	M	E
RI26	F	P	±16	No	No	0	RF6	F	P
RI27	F	P	±12	No	No	2	RF7	F	P
RI28	F	P	?	No	No	0	RF8	F	E
RI29	F	E	?	No	No	4	*Focus group 2: experts*		
RI30	F	E	±8	Yes	Yes	4	RF9	F	Researcher in public health
RI31	M	E	±10	No	No	4	RF10	M	Entrepreneur/consultant
RI32	F	E	0	Yes	No	1	RF11	F	Researcher in public health
RI33	F	E	±4	Yes	Yes	3	RF12	F	Entrepreneur/consultant
RI34	M	E	?	No	No	1	RF13	F	Entrepreneur/consultant
RI35	F	E	±24	No	No	4	*Additional interviews (professionals and experts)*		
							RI11	F	E
							RI12	F	E
							RI13	F	Researcher in public health
							RI14	F	E

^*∗*^F, female and M, male; ^*∗∗*^P, programme manager and E, epidemiologist.

**Table 2:** Findings of this study described as factors in their preferred end state.

Evaluation determinants	Programme managers/epidemiologists^*∗*^

Knowledge and skills	(+) Knowledge and experience with evaluation (−) Limited knowledge and experience with evaluation of ICIAs (−) Limited understanding of terminology (monitoring vs evaluation vs research) (−) Limited knowledge and experience with process evaluation (−) Poor knowledge of where to find evaluation support (−) Unaware of need for evaluation support of ICIA (+) Understanding that external parties need to be involved to collaboratively set evaluation goals (+) Knowledge about the relevance of making agreements about who would actually implement and evaluate these goals (+/−) Awareness of the need to communicate about evaluation towards stakeholders, but not always investing in such communication (−) Not aware evaluation needs to be managed

Support and finance	(+) Availability and good collaboration with an epidemiologist for evaluation expertise and responsibility for evaluation (−) Scarcity of time to conduct comprehensive evaluations (+/−) Availability of students, volunteers, coordinators, public and private partners to collect data (−) Difficulty of obtaining data from certain target groups (+/−) External partners having a stake in evaluation and motivated to conduct one (−) Stakeholders not knowing the JOGG-approach

Motivation	(−) Not considering it their task to evaluate their ICIA (−) Limited participation in the evaluation trainings and meetings offered by the JOGG office (+) Interested in evaluation since it could be used to improve the JOGG approach and achieve their goals (+/−) Feeling capable of conducting an evaluation after initial experience with it (+) Combined personal interest in evaluation and the topic of childhood obesity prevention and ICIAs (+) Opportunity to present the results on a national level and compare progress with other municipalities (+/−) Municipal interest in effect evaluation, but not in process evaluation (+) Evaluation as natural part of the work process (+) Guidance from an evaluation expert (coach or trainer) (−) A comprehensive evaluation manual, perceived to be in-compatible with available resources

^*∗*^A factor functions as a barrier (−) when it is not yet in place, it functions as a facilitator (+) when it is already in place and as an uncertain factor (+/−) when it is in place to some extent or if it sometimes functions as a barrier and sometimes as a facilitator.
