# Supplementary material for: Recommendations and Improvements for the Evaluation of Integrated Community-Wide Interventions Approaches
Source: J Obes. 2016 Dec 26;2016:2385698. doi: 10.1155/2016/2385698 (PMC5220506; doi:10.1155/2016/2385698)
Supplement: Supplementary file 1 — The Supplementary Material comprises of two instruments used for data collection and two developed tools for the analysis. Supplementary Data 1 is the interview guide for the semi-structured interviews with operationalized concepts from Michie's Behaviour Change Wheel [49]. Supplementary Data 2 is the topic list for the focus groups and additional interviews regarding the Evaluation Manual, based upon the innovation characteristics of Roger's Diffusion of Innovation Theory [50, 53]. Supplementary Data 3 is the code tree for the axial coding of the fragments of the semi-structured interviews and Supplementary Data 4 is the code tree for the axial coding of the fragments of the Focus Groups. [file 2385698.f1.pdf]

SUPPLEMENTARY MATERIAL SECTION TO THE FOLLOWING PAPER:

Recommendations and Improvements for the Evaluation of Integrated Community-wide Interventions Approaches.

Tessa M. van Koperen[a], Carry M. Renders[a], Eline J.M. Spierings[a], Anna-Marie Hendriks[c], Marjan J. Westerman[a], Jacob C. Seidell[a], Albertine J. Schuit[a,b]

Correspondence to: Marije van Koperen, VU University FALW, De Boelelaan 1085, 1081 HV Amsterdam, The Netherlands; E-mail: marije@cuprifere.nl; Telephone:#31-20-5983718

Supplementary 1: Interview guide for semi-structured interviews with operationalized concepts from ''The Behaviour Change Wheel'' [1]

1. Introduction (with informed consent)

2. Opportunities

- What are prohibiting factors for the evaluation?
- What are promoting factors?
- Is there sufficient budget to evaluate the JOGG-approach?
- What was your role in budget generating/ allocating (money, resources, personnel) for evaluation? Do you think that your role should have been done by someone else? If you could do it again, would you do it differently?
- Has anyone helped you in generating budget / resources?
- What do you think that your colleague's think of the evaluation of the JOGG-approach?
- Is there anyone in the organization that supports or stimulates the evaluation? Can you tell us more about that? Who is it and how does that person motivate?
- How did you experience the evaluation training and evaluation manual offered by the JOGG-office?
- How can evaluation be improved in the future?
- How could you help to better carry out the evaluation?

3. Motivation

- What is your view on the evaluation of the JOGG-approach?
- How do you feel / what is your opinion about the assessment handbook for and during the evaluation of the JOGG-approach?
- How do you feel / what is your opinion about the assignments between training sessions?
- What do you think would happen if the JOGG-approach is not evaluated?
- Have you previously evaluated such a large program, or played a part in it? How have you experienced that?
- When you evaluate does your opinion on the evaluation of the JOGG-approach play a role? Why / why not?
- How does 'priority' play a role in the evaluation of the JOGG-approach?
- To what extent do you feel responsible for evaluating the JOGG-approach? Why / why not?

4. Capability

- How should you evaluate the JOGG-approach?
- How do you evaluate the JOGG-approach according to the evaluation manual?
- To what extent do you have the capacity to carry out the evaluation process in satisfaction?
- How easy or how difficult is it for you to evaluate the JOGG-approach?
- To what extent do you know what is expected of you within the evaluation process of the JOGG-approach?
- How can you keep a good overview during the evaluation of the JOGG-approach?
- To what extent do you manage to understand the needs of different stakeholders involved in the JOGG-approach?
- How could you be supported to carry out the evaluation?

5. Closing

Supplementary 2: Topic list for the focus groups and additional interviews regarding the EM based upon the innovation characteristics of the Roger's Diffusion of Innovation theory [2]

Theme	**Topics**
Introduction	Introduction & Informed consent
	Goal clarification
Knowledge	Knowledge and awareness of EM*
Relative advantage	Opinion on EM*
	Opinion on additional tools in EM*
	Any (dis-)advantages of EM*
Compatibility	Usability of EM* in practice
	Perceived designated user
	Starting point of use
	Description of use
	Experiences and expectations of use
	Alignment of EM* use with programme budget
	Compatibility of EM* with organisational policy regarding evaluation
	Risks or negative consequences for organisation in step-wise use of EM*
	Personal reservations for step-wise use of EM*
	Missing information/ necessary add-ins
Attainability	For non-users: reservations for use (prohibiting factors)
	Necessary factors to stimulate use
Complexity	Complexity in use
	Opinion on offered evaluation method in EM*
	Suggestions for improvements to stimulate use
Lay-out	Opinion on lay-out
	Suggestions on lay-out improvements

* EM = Evaluation Manual

Supplementary 3: The code tree for the axial coding of the fragments of the semi-structured interviews

Evaluation

Context

- *Profession*
- *ICIA Implementation*

Evaluation Performance

- *Outcome evaluation*
- *Process Evaluation*
- *Collaboration evaluation*

Capability for evaluation of ICIA

- *Communication*
  - To inform
  - To discuss
- *Knowledge*
  - Evaluation design
  - Evaluation execution
  - Supportive means
- *Experience*
- Role in evaluation
  - distribution of roles and tasks
  - Manage, execute, inform
  - role responsibility
- *Understanding of*
  - Evaluation design
  - Evaluation execution
  - Relevance
- *Need for support*
  - Knowledge
  - Resources
- *Evaluation execution*
  - Generate time and budget
  - Setting goals
  - Determine feasibility
  - Involvement of stakeholders
  - Evaluation plan
- *Collaboration skills*

Motivation towards evaluation of ICIA

- *Attitude*
  - Evaluation in general
  - Responsibility
  - Relevance
  - Insight into own role
  - Evaluation capability
  - Knowledge
  - Experience
  - Costs
  - Magnitude of workload
- *Evaluation goal*
- *Usability of evaluation results*
- *Feasibility of evaluation*
- *Supportive means*
  - Evaluation Manual
  - Evaluation training
- *Priority*

Opportunity to conduct evaluation of ICIA

- *Available capacity*
  - Generated budget
  - Manpower
  - Students
- *Stakeholders*
  - Time available
  - Role specification
  - Capability
  - Priority
  - Pressure/demand/ support
  - Collaboration
  - Communication
  - Provision of budget
  - Willingness to participate
  - Understanding JOGG-approach
  - Interest in evaluation
- *Supportive factors*
  - Resources
  - JOGG evaluation expert
  - Evaluation Manual
  - Evaluation training
- *Target group*
  - Accessibility
  - Privacy
- *Implementation JOGG*
  - Development
  - Complexity
  - Number of activities

Collaboration between municipalities
  - Knowledge sharing
  - Resources sharing

Supplementary 4: The code tree for the axial coding of the fragments of the Focus Groups

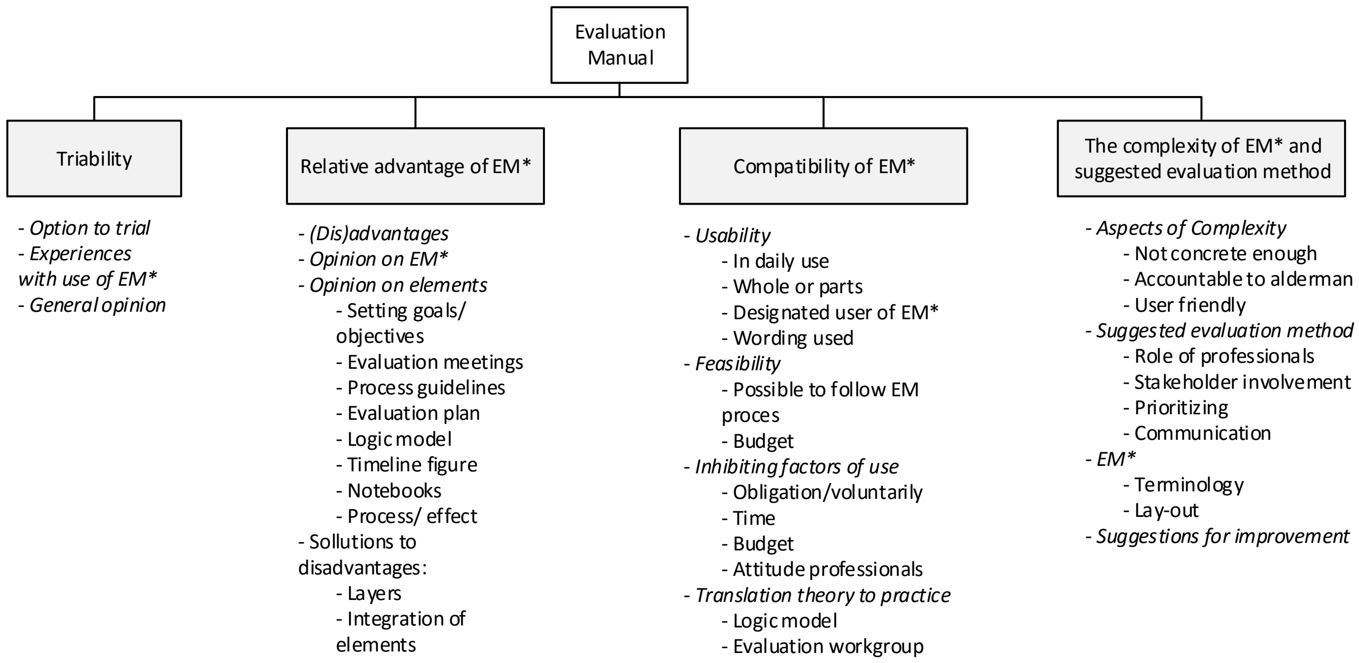

5

*EM = Evaluation Manual

References

1. Michie, S., M. van Stralen, and R. West, *The behaviour change wheel: A new method for characterising and designing behaviour change interventions.* Implementation Science, 2011. **6**(1): p. 42.
2. Rogers, E.M., *Diffusion of preventive innovations.* Addictive Behaviors, 2002. **27**(6): p. 989-993.
